# Combined Epidermal Growth Factor Receptor and Beclin1 Autophagic Protein Expression Analysis Identifies Different Clinical Presentations, Responses to Chemo- and Radiotherapy, and Prognosis in Glioblastoma

**DOI:** 10.1155/2015/208076

**Published:** 2015-03-03

**Authors:** Paolo Tini, Giuseppe Belmonte, Marzia Toscano, Clelia Miracco, Silvia Palumbo, Pierpaolo Pastina, Giuseppe Battaglia, Valerio Nardone, Marie Aimée Gloria Munezero Butorano, Armando Masucci, Alfonso Cerase, Luigi Pirtoli

**Affiliations:** ^1^Tuscan Tumor Institute (ITT), 50139 Florence, Italy; ^2^Unit of Radiation Oncology, University Hospital of Siena, 53100 Siena, Italy; ^3^Department of Medicine, Surgery and Neurological Sciences, Research Center for Molecular Radiobiology, University of Siena, 53100 Siena, Italy; ^4^Unit of Pathological Anatomy, Department of Medicine, Surgery and Neurological Sciences, University of Siena, 53100 Siena, Italy; ^5^Department of Biology and Biotechnology, University of Pavia, 27100 Pavia, Italy; ^6^Unit of Preventive Medicine, Moscati Hospital, 83100 Avellino, Italy; ^7^Unit of Neuroradiology, University Hospital of Siena, 53100 Siena, Italy

## Abstract

Dysregulated EGFR in glioblastoma may inactivate the key autophagy protein Beclin1. Each of high EGFR and low Beclin1 protein expression, independently, has been associated with tumor progression and poor prognosis. High (H) compared to low (L) expression of EGFR and Beclin1 is here correlated with main clinical data in 117 patients after chemo- and radiotherapy. H-EGFR correlated with low Karnofsky performance and worse neurological performance status, higher incidence of synchronous multifocality, poor radiological evidence of response, shorter progression disease-free (PDFS), and overall survival (OS). H-Beclin1 cases showed better Karnofsky performance status, higher incidence of objective response, longer PDFS, and OS. A mutual strengthening effect emerges in correlative power of stratified L-EGFR and H-Beclin1 expression with incidence of radiological response after treatment, unifocal disease, and better prognosis, thus identifying an even longer OS group (30 months median OS compared to 18 months in L-EGFR, 15 months in H-Beclin1, and 11 months in all GBs) (*P* = 0.0001). Combined L-EGFR + H-Beclin1 expression may represent a biomarker in identifying relatively favorable clinical presentations and prognosis, thus envisaging possible EGFR/Beclin1-targeted therapies.

## 1. Introduction

Glioblastoma (GB) is the most frequent primary brain neoplasm and one of the most lethal tumors. Standard treatment is the maximal safe surgical resection followed by adjuvant radiotherapy (RT) and chemotherapy (CHT) [1]. Despite this multimodal treatment, prognosis remains poor, with less than 5% of the patients alive beyond five years from diagnosis [2]. Extensive multiplatform genomic characterization is increasing our understanding of the molecular bases of GB and is leading to the discovery of promising novel therapeutic targets, although efficient new treatments are still not available [3–5].

*Epidermal growth factor receptor* (EGFR) gene mutations, amplification and overexpression, EGFR protein overexpression, and PI3K-Akt-mTOR-EGFR pathway dysregulation are hallmarks of GB, usually related to an aggressive phenotype [6] and characterize the most frequent GB molecular subtype showing the classical expression profile [7].

The PI3K-Akt-mTOR signaling pathway, driven or not by EGFR activation, negatively regulates autophagy [8]. Autophagy is a degrading, self-eating cellular process involved in an array of physiological and paraphysiological cellular functions [9]. Its relevance is emerging also in cancer, in which, based on cell context, tumor type, and stage, autophagy may play different roles [10]. While autophagy halts tumor initiation, in advanced cancer it can either promote tumor progression, allowing cell survival, or lead to cell death [11]. Autophagy-related death, also known as “type II programmed cell death,” has been recognized as a major type of nonapoptotic death in GB, both* in vivo* [12] and* in vitro* [13], being induced by RT and CHT [14, 15]. Thus, novel autophagy-based GB treatment approaches may be envisaged. Therapeutic perspectives also derive from the complex crosstalk between autophagy genes and apoptotic and other types of cell death [16, 17]. The autophagic gene* Beclin1* and its complex with either Bcl2 or Vps34, the Class III PIK involved in autophagosome initiation, are key determinants of autophagy and cell fate [18].

Beclin1 also binds EGFR [19] and EGFR is able to directly regulate Beclin1 and autophagy also in an mTOR-independent manner [19]. EGFR promotes tumor growth and cell motility [20] and has been associated with a poor clinical GB outcome and unfavorable GB presentation [21]. Beclin1 expression, instead, decreases with tumor progression [22, 23], and we observed also that it is positively correlated with a better GB patient clinical outcome [24]. In a recent study, we found that the modulation of autophagy and EGFR expression has an impact on GB cell migration activity and response to radiation treatment [25].

We are not aware of previous studies correlating EGFR and Beclin1 expression with clinical features in GB. Here, we retrospectively analyzed the potential relevance of concomitant Beclin1 and EGFR protein expression and examined their colocalization in a series of patients affected by GB, aiming at investigating clinical implications of the patterns of their expression in GB tissue, out of a patient series undergoing postoperative CHT-RT.

## 2. Materials and Methods

### 2.1. Ethics Approval

This study was approved by both the Institutional Review Board and Ethics Committee of the University Hospital of Siena, and all the patients had provided signed informed consent before any treatment.

### 2.2. Patients

We retrospectively reviewed the medical records of patients affected by GB (Grade IV-WHO Classification [26]) submitted to the Radiation Oncology Unit of the University Hospital of Siena for postoperative adjuvant CHT-RT from February 2002 to November 2013. Patients who had undergone a full-course RT and CHT (i.e., standard RT and concurrent and sequential temozolomide (TMZ) administration) were included in the study, whereas patients enrolled in clinical trials with experimental RT/CHT with antiangiogenetic, anti-EGFR, and any other targeted therapy were excluded.

Patients were referred, treated, and followed up on a three-month interval basis after therapy in our unit. All clinical and pathological data were recorded, including extent of surgery and histological diagnosis, clinical examination, blood counts and chemistry, Karnofsky performance status (KPS), neurological performance status (NPS), chest X-ray, and pre- and postoperative and follow-up magnetic resonance imaging (MRI).

### 2.3. Postoperative Treatment

RT and TMZ CHT schedules were adopted as previously described, according to a protocol-driven schedule [27]. Briefly, RT consisted of a 54–60 Gy total dose administered with three-dimensional conformal irradiation in all cases with a unifocal presentation. The planning target volume (PTV) [28] included residual tumor mass and postoperative cavity with a 2-3 cm margin. Suitable patients with small lesions received a boost dosage up to 70 Gy, limited to the gross tumor volume (GTV) [28] if no obvious progression or relevant toxicity occurred during the previous irradiation course. Patients with multifocal lesions were submitted to whole brain irradiation, up to 50 Gy. Five weekly 1.8–2 Gy sessions were administered during the entire RT course, in all cases. All patients received TMZ concurrent with RT (75 mg/mq/die) up to a maximum of 7 weeks, and most of them also received sequential TMZ CHT (150–200 mg/mq for 5 days, every 28 days), unless tumor progression or relevant toxicity occurred. The patients included in this evaluation completed at least 80% of the planned treatment.

### 2.4. Follow-Up

After treatment, all patients were included in a follow-up program. General and neurological examinations, with blood counts and chemistry, were performed every three months, as previously outlined.

### 2.5. Clinical Variables Included in the Study

Age: A cut-off value of 50 years (≤50 y, >50 y) was established according to literature [2].

KPS: Two categories were considered, 100–80%; ≤70%, after surgery.

NPS: Patients were assigned to five categories after surgery (1: no neurological impairment; 2: some neurological impairment; 3: moderate impairment; 4: major functional impairment; 5: no conscious response), according to the Medical Research Council Brain Tumor Working Party [29].

MRI disease presentation (unifocal versus synchronous multifocal disease) was assessed at the preoperative MRI examination. Multifocality consisted of at least two lesions at the gadolinium-enhanced T1 sequence, separated by a distance of at least 1 cm.

After RT and concurrent TMZ administration, MRI was repeated, in order to assess the subsequent tumor volume evolution with respect to pre-RT status, the first time at 2-3 weeks after completion, then on a 3-month basis, and in any case of suspicion of tumor progression on clinical grounds.

Radiological response (RR) was so detected and classified into complete response (CR), partial response (PR), stable disease (SD), and progressive disease (PD) at the first MRI examination. Objective response is defined as OR = CR + PR. RR was assessed using either MacDonald's criteria [30]or response assessment in neurooncology (RANO) criteria [31], respectively, before and after 2010.

### 2.6. Molecular Determination of the Methylation Status of the MGMT Gene Promoter

MGMT gene promoter methylation was assessed by methylation-specific polymerase chain reaction (PCR). Briefly, genomic DNA was extracted from paraffin-embedded tumor sections and treated with sodium bisulfite using the EZ DNA Methylation-Gold kit (HISS Diagnostics, GmbH, Freiburg, Germany). Primer sequences were used to detect methylated and unmethylated MGMT promoter sequences. PCR products were separated on 2% agarose gel. A glioma cell line, with a completely methylated MGMT promoter, and peripheral blood mononucleated cells served as positive and negative control samples, respectively.

### 2.7. EGFR and Beclin1 Immunohistochemistry

In each case, 3 *μ*m thick sections were cut from paraffin blocks of 10% formalin-fixed tumor fragments and processed for immunohistochemistry. Briefly, after deparaffinization and rehydration, before applying the anti-EGFR mouse monoclonal (clone EGFR.25, ready to use, and catalogue number: RTU-EGFR-384, Novocastra, Milan, Italy) or the anti-Beclin1 rabbit polyclonal (gene ID: 8678, amino acids 329–345, diluted 1 : 200, and catalogue number: B6186, Sigma-Aldrich, Milan) primary antibodies, sections were pretreated either with Pronase XIV of* Streptomyces griseus* (Bio-Optica, Milan, Italy) at 37°C for 10 minutes or with WCAP citrate buffer pH 6.0 (Bio-Optica), for 40 min at 98.5°C, respectively.

The evaluation of the signal was performed by UltraVision LP Large Volume Detection System HRP Polymer (Bio-Optica, Milan), with the diaminobenzidine chromogen (Dako) for 8 min. Sections were then counterstained with Meyer's hematoxylin. In all cases, negative controls were performed by repeating the procedure and omitting the primary antibody.

### 2.8. Assessment of Immunostaining

Staining was independently evaluated by two of the authors (CM, MAGMB), at medium resolution (20x objective, eye piece 1.25) all throughout tumor sections. EGFR membranous and/or cytoplasmic and Beclin1 cytoplasmic immunoreactivity scored 0 if negative and from 2 to 5, if positive, on the basis of both the stain's intensity (1: weak, 2: moderate, and 3: strong) and the percentage of positive cells (1: ≤50%, 2: >50%). We considered scores 0–2 as a low (L) and scores 3–5 as a high (H) protein expression, respectively.

### 2.9. Double EGFR-Beclin1 Immunofluorescence Stain

In order to colocalize EGFR and Beclin1 protein in tumor cells, in representative cases of each group of low or high protein expressing GBs, a double immunofluorescence stain was performed. Briefly, 4 *μ*m thick sections were deparaffinized in xylene and rehydrated in graded ethanol solutions (100%, 95%, 80%, and 70%), 5 minutes each, and washed in dH_2_O. Then, antigen retrieval was obtained by incubation with 10 mM sodium citrate buffer (pH 6.0) at a subboiling temperature for 20 min. Sections were then cooled for 10 min, washed in phosphate-buffered saline (PBS), and incubated overnight at 4°C with the following antibodies: mouse anti-EGFR (Undiluted, Novocastra); rabbit anti-Beclin1 (diluted 1 : 200, Sigma-Aldrich). The slides were washed three times with PBS and incubated with the secondary antibody fluorochrome conjugate (goat anti-rabbit Alexa Fluor 488, goat anti-mouse Alexa Fluor 568) for 1 hour at room temperature in the dark. The nuclei were counterstained by incubating the sections for 10 min with 4′,6-diamidino-2-phenylindole (DAPI). Slides were washed in PBS and mounted with Antifade. In each case, a negative control was generated by omitting the primary antibody. Images were acquired and analyzed with a microscope Leica AF CTR6500HS (Microsystems).

### 2.10. Western Blotting

Tissue samples were lysed in radioimmunoprecipitation assay (RIPA) buffer containing protease inhibitors, following standard procedures. After protein determination, using BioRad protein assay (BioRad, Milan, Italy), equal amounts of proteins (40 *μ*g) were resolved on 8% SDS-PAGE gel and transferred to a nitrocellulose membrane (BioRad). Membranes were blocked with 3% nonfat milk (BioRad) in PBS Tween 0.05% (PBST) and incubated overnight at 4°C with the following antibodies: anti-EGFR (undiluted, Novocastra), anti-Beclin1 (1 : 270, Sigma-Aldrich), and anti-*β*-actin (1 : 500, catalogue number: 04-1116, MERCK Millipore Corporation, Billerica, MA, US) diluted with 3% nonfat milk in PBST. Membranes were washed three times in PBS Tween 0.1% and incubated with specific secondary antibodies diluted with 3% nonfat milk in PBST (goat anti-rabbit IgG (H + L)-HRP conjugate, diluted 1 : 10000, catalogue number: 172-1019, BioRad; goat anti-mouse IgG (H + L)-HRP conjugate, diluted 1 : 5000, catalogue number: 172-1011, BioRad) for 1 h at RT. The membranes were incubated with ECL reagents (BioRad) for 1 min and then were developed on Hyperfilm ECL (Amersham GE Healthcare, 28906835).

Images of the bands were digitized and the densitometry was performed using the open source image processing program ImageJ (http://imagej.nih.gov/ij/); *β*-actin bands were used for normalization.

### 2.11. Statistical Analysis

We performed a correlation analysis using Spearman's rho two-tailed correlation test between clinical parameters and L- and H-EGFR/Beclin1-expression groups. Overall and progressive disease-free survival (OS and PDFS, resp.) were calculated with the Kaplan-Meier method. The univariate survival analysis was used to identify prognostic parameters as follows: clinical factors (age, KPS, NPS, and synchronous multifocality), treatment-related factors (extent of surgery, RT dose, sequential TMZ, and radiological response from MRI scans), and biological factors (EGFR/Beclin1 protein expression).

We used the log-rank test to assess the significance of survival differences for the considered parameters (*P* values ≤ 0.05 were considered as statistically significant). We also performed a multivariate analysis (Cox regression) to quantify the relationship between survival and potential predictors, in order to identify a subgroup of independent factors significantly related to survival. All statistical analyses were performed with the SPSS 15.0 software package for Windows.

## 3. Results

### 3.1. Patients and Postoperative Treatment

**Figure 1:**
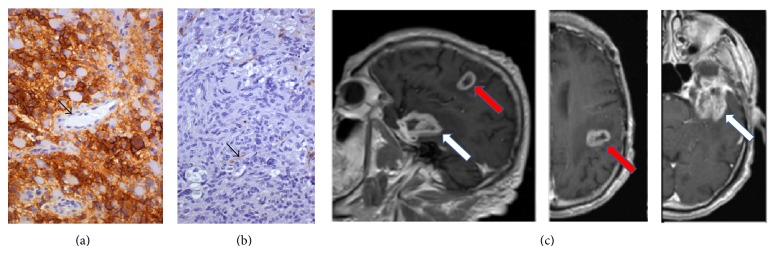
Patient with H-EGFR (a) and L-Beclin1 (b) pattern of protein expression in a GB. The arrows indicate the endothelium of vessels, negative for EGFR (a) and positive for Beclin1 (b) Immunohistochemistry, diaminobenzidine, original magnification ×200. The MRI T1-sequence with gadolinium (c) shows a multifocal GB with a left temporal (white arrow) and a parietooccipital lesion (red arrow).

One hundred and seventeen patients were included in this study, all planned for RT and concurrent TMZ. At surgery, 23 had a gross tumor resection (GTR) (19.7%) and 94 (80.3%) a biopsy or a subtotal tumor resection (B-STR), respectively. Median age was 62 years (range 26–83), 15 patients (12.8%) were ≤ 50 y, and the other 102 were >50 y (87.2%). The KPS score was 100–80 in 91 patients (77.8%) and ≤ 70 in 26 (22.2%). The NPS score, at admission, was 1-2 in 61 patients (52.1%), 3 in 34 patients (29.1%), and 4 in 22 patients (18.8%). Preoperative MRI showed a single tumor in 98 out of 117 patients (83.8%) and a multifocal presentation in 16 patients (16.2%) (Figure 1(c)).

Forty-three patients (36.6%) received a RT dose of <54 Gy (due to a multifocal presentation or constraints, such as critical structures very close to the tumor), 47 (33.8%) a dose of 54–60 Gy, and 27 (23.7%) a boost up to 70 Gy, according to the aforementioned protocol. Ninety-one (77.6%) patients completed the full-course of TMZ concurrently to RT; out of them, sequential TMZ was then administered in 76 patients (64.9%), until the events of tumor progression or severe toxicity (64.9%), whereas 41 (35.1%) patients did not have this treatment scheduled, due to early tumor progression or toxicity at the end of the concurrent RT and TMZ administration.

### 3.2. Results of Treatment

After RT and concurrent TMZ, the RR demonstrated that 19 patients (16.2%) had a CR, 26 (22.2%) a PR (thus an OR was achieved in 45 patients, 38.5%), and 25 (21.4%) a SD, whereas 47 (40.2%) experienced a PR at the post-RT MRI controls. The median OS was 11 months; 1-year and 2-year OS values were, respectively, 47.8% and 25.4%. The median PDFS was 10 months (39.6% at 1 year and 27.0% at 2 years, resp.).

### 3.3. Methylation Status of MGMT Gene Promoter

MGMT methylation status was assessed in 83 patients: the MGMT promoter was unmethylated in 45 cases (54.2%) and methylated in 38 (45.8%).

### 3.4. EGFR and Beclin1 Immunohistochemistry

**Figure 2:**
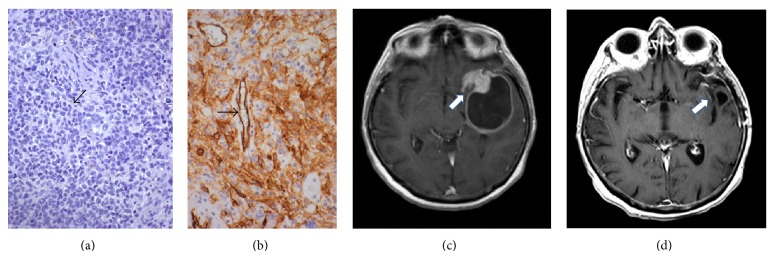
L-EGFR (a) and H-Beclin1 (b) patterns of protein expression in a GB. The arrows indicate the endothelium of vessels, negative for EGFR (a) and positive for Beclin1 (b) Immunohistochemistry, diaminobenzidine, original magnification ×200. (c) MRI T1-sequence with gadolinium shows a left temporal glioblastoma (white arrow) before treatment. (d) Radiological complete response (white arrow) after treatment (MRI at 4 months after adjuvant RT-CHT treatment).

**Figure 3:**
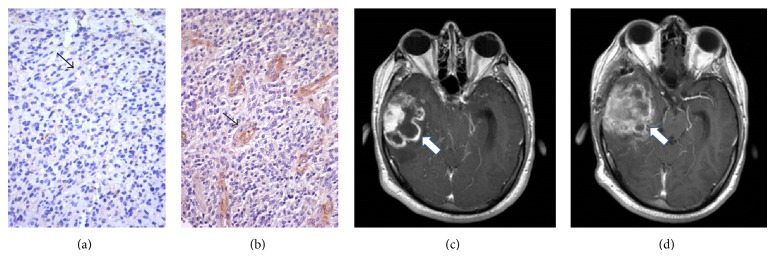
Patient with L-EGFR (a) and L-Beclin1 (b) pattern of protein expression in GB. The arrows indicate the endothelium of vessels, negative for EGFR (a) and positive for Beclin1 (b) Immunohistochemistry, diaminobenzidine, original magnification ×200. (c) MRI T1-sequence with gadolinium shows a right temporal lesion (white arrow) before treatment. (d) Radiological progression (white arrow) after treatment (MRI at 6 months after adjuvant RT-CHT treatment).

EGFR membranous and/or cytoplasmic positivity was observed in most GBs, while few nuclei were decorated by EGFR in a minority of cases. Some cases were completely negative for EGFR. EGFR staining was not observed in vessel endothelia or in normal/reactive glia (Figures 1(a), 2(a), and 3(a)). On the other hand, Beclin1 stained normal cells and was heterogeneous in GBs, with a higher number of cases negative or lowly expressing the protein; a pin point cytoplasmic staining and variable nuclear immunopositivity were observed (Figures 1(b), 2(b), and 3(b)). In several cases, a heterogeneous expression of both proteins was observed in limited areas.

### 3.5. Assessment of Immunostaining Patterns and Double EGFR-Beclin1 Immunofluorescence Stain

There were 68 cases (58%) expressing H-EGFR; 49 (41.7%) expressing L-EGFR; 59 (50.4%) expressing L-Beclin1, and 58 (49.6%) expressing H-Beclin1.

**Figure 4:**
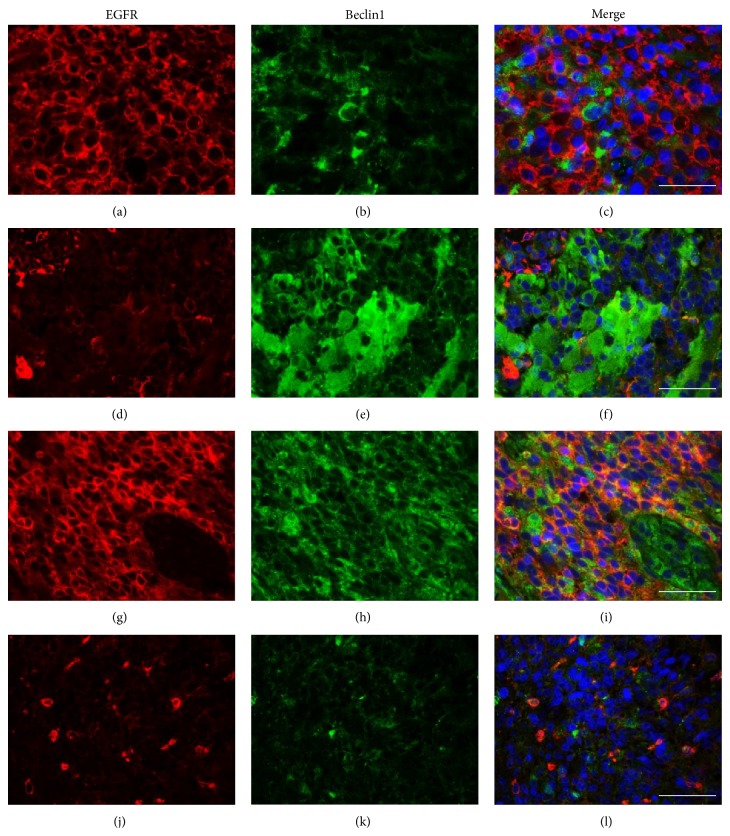
Double immunofluorescence staining for EGFR (a, d, g, and j; red stain) and Beclin1 (b, e, h, and k; green stain). Representative GBs showing H-EGFR + L-Beclin1 (a, b, and c), L-EGFR + H-Beclin1 (d, e, and f), H-EGFR + H-Beclin1 (g, h, and i), and L-EGFR + L-Beclin1 (j, k, and l) patterns of protein expression. Nuclei are marked by staining with 4,6-diamidino-2-phenylindole (DAPI) and appear blue in the merged pictures (c, f, i, and l). Original magnification ×650. Scale bar = 50 *μ*m.

**Figure 5:**
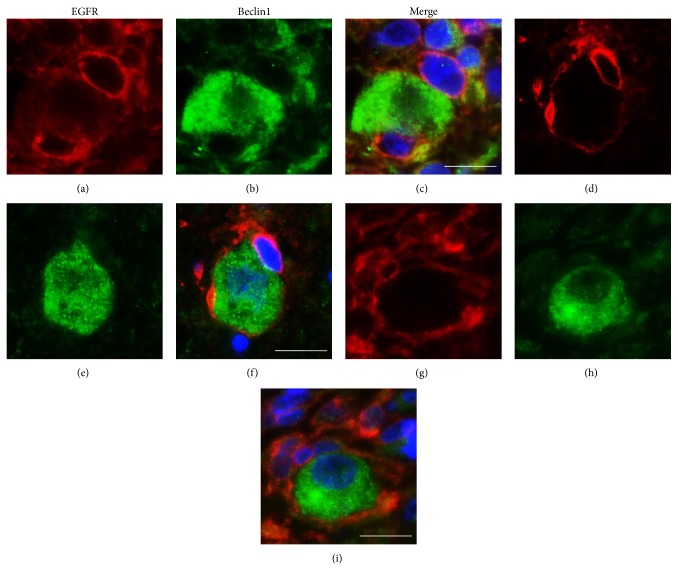
Double immunofluorescence staining. An admixture of H-EGFR (a, d, and g; red stain) and H-Beclin1 (b, e, and h; green stain) positive GB cells is observable in areas showing a heterogeneous pattern of protein expression. The merged pictures (c, f, and i), however, show that the positivity is mutually exclusive; a pin point cytoplasmic positivity for Beclin1 is evident in these enlarged details of Figure 2. Scale bar = 25 *μ*m.

Overall, two main immunoreactivity patterns were observable: H-EGFR/L-Beclin1 (34 cases, 29.1%; Figures 1, 4(a), 4(b), and 4(c)) and L-EGFR/H-Beclin1 (24 cases, 20.5%; Figures 2, 4(d), 4(e), and 4(f)), the former being the dominant pattern in the majority of cases, although there was a large stain heterogeneity. In fact, there were also cases either highly (H-EGFR/H-Beclin1; Figures 4(g), 4(h), and 4(i)) or lowly expressing (L-EGFR/L-Beclin1; Figures 3, 4(j), 4(k), and 4(l)) the two proteins. Furthermore, several cases showed heterogeneous expression of both proteins in limited areas (Figure 5). EGFR and Beclin1 protein expressions were mutually exclusive in large areas in many cases. This was more evidenced by the double EGFR-Beclin1 immunofluorescence stain (Figure 5).

### 3.6. Western Blotting

**Figure 6:**
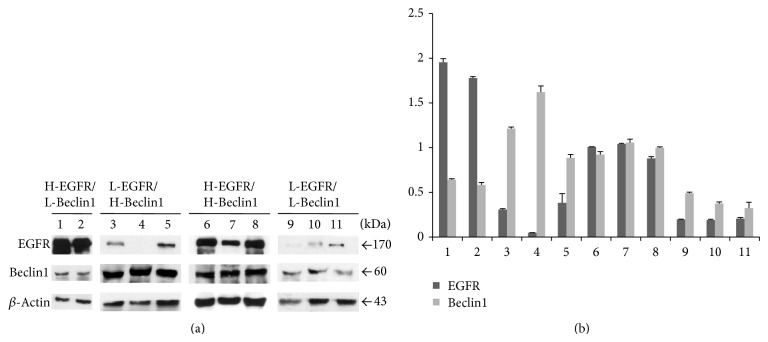
(a) Western blotting for EGFR (170 kDa) and Beclin1 (60 kDa) in representative H-EGFR/L-Beclin1 (lanes 1 and 2), L-EGFR/H-Beclin1 (lanes 3–5), H-EGFR/H-Beclin1 (lanes 6–8), and L-EGFR/L-Beclin1 (lanes 9–11) GBs. (b) Up- or downregulation of either EGFR or Beclin1 is quantified by densitometric data analysis, which shows the relative expression of EGFR and Beclin1 after normalization to the *β*-actin bands. Data are reported as means ± S.E. of three densitometric analyses of the same sample.

Specific EGFR (170 kDa) and Beclin1 (60 kDa) bands were detected and high versus low protein expression was confirmed in several cases, representative of H-EGFR/L-Beclin1, L-EGFR/H-Beclin1, H-EGFR/H-Beclin1, and L-EGFR/L-Becin1 groups of patients (Figure 6).

### 3.7. Statistics

#### 3.7.1. Univariate Analysis

Distribution of EGFR and Beclin1 protein expression according to age, KPS, NPS, RMN disease presentation, extent of surgery, data regarding local tumor control, and significant *P* values are shown in Table 1.

PDFS and OS were negatively correlated with age (*P* = 0.035 and 0.04, resp.), KPS (*P* = 0.001), NPS (*P* = 0.0001), synchronous multifocality at preoperative MRI (*P* = 0.001), extent of surgical resection (*P* = 0.0001 and 0.001, resp.), radiation dose (*P* = 0.04 and 0.033, resp.), and sequential TMZ (*P* = 0.0001 and 0.001, resp.) (Table 2).

**Figure 7:**
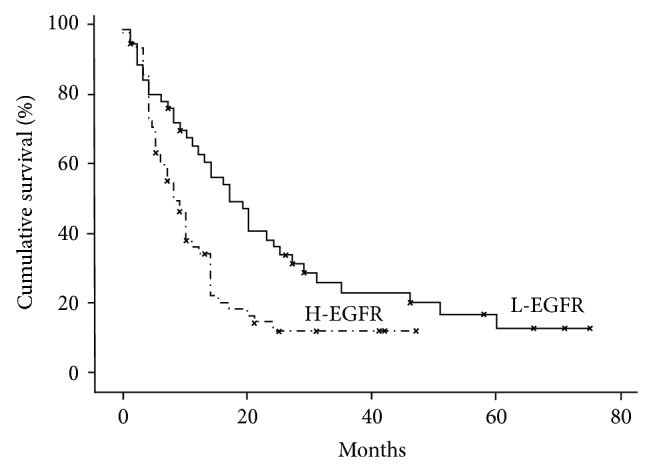
Kaplan-Meier survival curves for EGFR expression levels (*P* value < 0.05).

EGFR and Beclin1 expressions were not correlated with each other. H-EGFR significantly correlated with low KPS (*P* = 0.03), a worse NPS class (*P* = 0.002), a synchronous multifocal presentation (*P* = 0.01) (Figure 1(c)), a worse RR (*P* = 0.013), a shorter PDFS (*P* = 0.002), and OS (*P* = 0.004). H-EGFR versus L-EGFR patients had, in fact, a worse median PDFS (5 months versus 14 months) and OS (9 months versus 18 months) (Figure 7).

H-Beclin1 was instead positively correlated with a better KPS (*P* = 0.009), a higher OR (*P* = 0.002), and a better PDFS and OS (*P* = 0.001).

**Figure 8:**
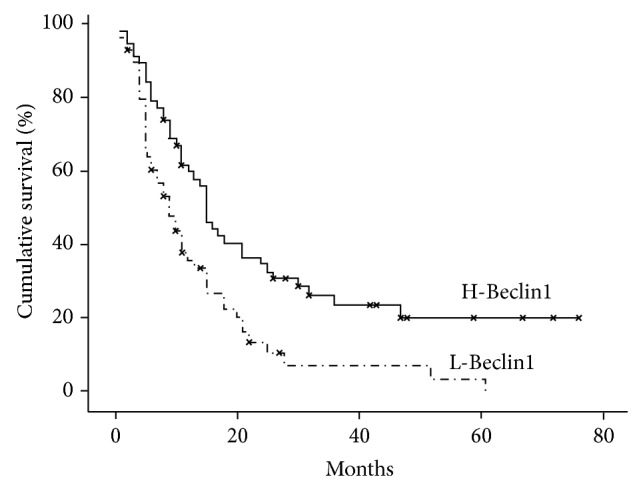
Kaplan-Meier survival curves for Beclin1 expression levels (*P* value < 0.05).

H-Beclin1 patients had a median OS of 15 months, compared to 5 months for the L-Beclin1 group (Figure 8). Clustering L-EGFR and H-Beclin1 expression resulted in a stronger correlation with a better RR (*P* = 0.001) (Figures 2(c) and 2(d)) than other patterns of expression (Figures 3(c) and 3(d)) and absence of MRI multifocality of disease at onset (*P* = 0.002). In particular, no multifocal disease was found in this subgroup of patients.

**Figure 9:**
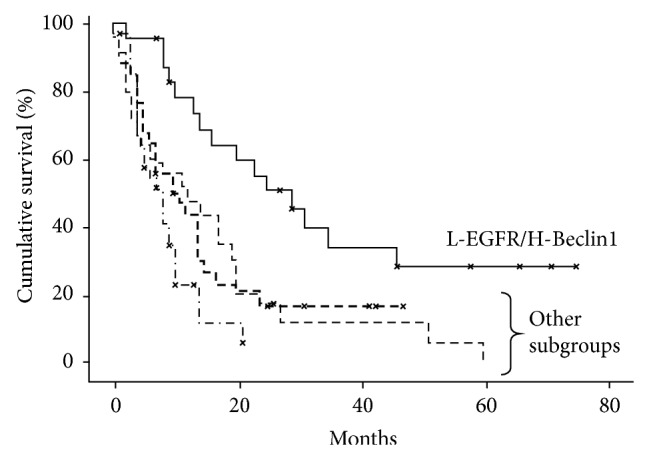
Kaplan-Meier survival curves for EGFR and Beclin1 coexpression (*P* value < 0.05).

Clustering EGFR expression and Beclin1, we found that H-Beclin1/L-EGFR had a significantly better prognosis, with a median survival of 30 months, compared to 18 months of L-EGFR, 15 months of H-Beclin1, and 11 months of all GBs (Figure 9).

#### 3.7.2. Cox Regression Proportional Hazards Regression Multivariate Analysis

Given the dependency of the clinical/therapeutic factors on the relationship with EGFR and Beclin1 expression (Table 1), the multivariate analysis of survival factors evidenced that H-EGFR (HR: 2.21; 95% CI: 1.404–3.481; *P* = 0.001), L-Beclin1 (HR: 1,898; 95% CI: 1.244–2.896; *P* = 0.003), and B-STR (HR: 3,119; CI: 1.711–5.669; *P* = 0.0001) were independently associated with a shorter survival.

## 4. Discussion

Glioblastoma has a poor prognosis. Patients under current therapies have a median survival of approximately one year after diagnosis, and it rarely exceeds 15 months for patients enrolled in clinical trials [5]. An increased efficacy of the standard of care was recently achieved, in fact, with TMZ concomitant and adjuvant in respect to radiotherapy [32, 33]. However, response to therapy and prognosis highly depends on both clinical and molecular determinants. Clinical trials as well as retrospective case series analyses outline the prognostic profile of subgroups of patients with a more favorable outcome, based on classical factors (i.e., young age, a good KPS and NPS, circumscribed neoplasms arising in noneloquent areas allowing for a gross total resection, and a high RT dose, i.e., 60 Gy [2, 34]), which were all associated with a better prognosis also in our series, novel chemoradiotherapeutic approaches [33], and molecular biomarkers (i.e., themethylation of O6-methylguanine-DNA methyltransferase (MGMT) gene promoter [32] and mutations of* isocitrate dehydrogenase (IDH1)* gene [35]). We also found a significant correlation of MGMT methylation with a longer survival, although the analysis was not conducted in all the cases. The network of prognostic factors is continuously growing and large-scale genome analyses are further subgrouping molecular subtypes of GB associated with different prognosis [3, 7]. However, to date, among molecular biomarkers, only the MGMT promoter methylation status predictive biomarker has an undoubtedly high impact on clinical practice, being used for stratification of RT and TMZ-CHT treatment regimes, leading to a limited but significant improvement of survival [32, 34].

Therefore, further molecular prognostic biomarkers and targets for identifying patients at a higher prognostic risk are needed, for enlarging the horizon of future individualized therapies.

Our study suggests that the combined evaluation of EGFR and Beclin1 autophagic protein expression in tumor tissue sections could add valuable information to the prognostic molecular profile of GB.

In our experience, in fact, multivariate analysis indicated that, combining the two variables, that is, L-EGFR and H-Beclin1 expression, a subgroup of patients (20.4%) with a more favorable prognosis could be identified. This subgroup of GB patients reached a median survival of 30 months compared to 11 months of all the 117 cases, 18 months of L-EGFR, and 15 months of H-Beclin1 protein expressing GB subgroups. Furthermore, EGFR/Beclin1 protein expression identified subgroups of GB patients with a different clinical presentation, in terms of clinical and neurological patient status and multifocality of lesions and edema, and with MRI radiological evidence of response to therapy, in terms of ORs. The worst clinical set was associated with a H-EGFR and the best with a L-EGFR + H-Beclin1 protein expression profile. In the latter group, in particular, in no case was there multifocality of the neoplasm at onset.

EGFR protein overexpression and gene mutation/amplification are known drivers of gliomagenesis and GB aggressiveness, activating signaling cascades that trigger tumor cell proliferation and invasiveness, angiogenesis, and suppressing apoptotic cell death [20, 36–39], largely contributing to the high RT-CHT therapy resistant GB phenotype [6, 39, 40]. EGFR alterations are found in most GBs and characterize the most frequent molecular classic subtype [3], thus being an ideal targetable molecule. However, the use of monoclonal antibodies for therapy has not yielded promising results to date in clinical trials [41]. Novel strategies are intended to target the key phosphorylated kinases and/or altered metabolic pathways downstream EGFR [41]. Autophagy is one of the altered metabolic pathways inhibited by EGFR, which acts via mTOR [8] or by direct inhibition of Beclin1, a cytoplasmic protein that induces autophagy by binding to Vps34-Vps15 core [8, 18, 19, 42]. It is known that this catabolic process acts as a tumor suppressor in the early phases of carcinogenesis, while, in advanced neoplasms, depending on tumor cell context and type, it may either promote or inhibit cancer progression and therapy resistance, being also able to induce an autophagy-related or type II programmed tumor cell death [11]. The authors [22], as others [23], found that Beclin1 was underexpressed in most GBs: this was associated with a decreased apoptosis and negatively impacted on prognosis [24]. The overall negative impact of activated EGFR on autophagy could partly explain the alternate, almost mutually exclusive expression of EGFR and Beclin1 that we observed in most GBs. By examining the colocalization of the two proteins with a double immunofluorescence stain, in areas showing heterogenous protein expression, cells positive for EGFR were negative for Beclin1 and vice versa.

EGFR's direct inhibition of Beclin1 and/or the existence of mutated forms of Beclin1 recently observed in human nonsmall cell lung carcinoma cells [19], in which EGFR was found colocalized with Beclin1 in autophagic vacuoles, support the coexpression of the two proteins that we also observed in GB cells. The distribution of the two proteins was, in fact, heterogenous in many cases, although subgroups of GBs could be identified based on the dominant protein expression pattern. Other unknown-to-us factors may occur in cases in which both proteins were negative. Synchronous multifocality of newly diagnosed GBs and GB aggressiveness are partly linked to the invasion ability of neoplastic cells, resulting from an orchestrated activation of cell migration, in which EGFR plays a pivotal role [38, 43–45].

Low expression of Beclin1 was also related to distant metastasis risk in breast cancer [46] and recently we demonstrated that the combined silencing of EGFR and induction of autophagy by rapamycin has additive effects both in increasing radiation sensitivity and in inhibiting cell migration ability, in U373 and T98G GB cells [25]. Therefore, both L-EGFR and H-Beclin1 might have interacted in contributing to the minor aggressiveness, the higher MRI evaluated response to therapy, and the lack of multifocal presentation, which we observed in the GB group with a more favorable prognosis, whereas the leading EGFR ability on cellular invasiveness may partly justify why H-EGFR patients showed a significantly higher multifocal presentation, besides a more frequent progression of the disease at imaging after treatment and a poorer prognosis.

MGMT methylation was positively correlated with prognosis. However, it was not correlated with either EGFR or Beclin1 expression, further supporting their independent role as prognosis biomarkers, despite the fact that MGMT analysis was not performed in all our GB cases.

## 5. Conclusion

Our results provide a preliminary assessment of the role of EGFR and Beclin1, extrapolated from a series of GB patients treated according to a prospectively definite protocol. Some correlations, in terms of clinical features at referral, imaging data, response to RT-TMZ treatment, and survival, can be established with EGFR and Beclin1 expression. In particular, some parameters of aggressiveness in GBs, such as multifocality (probably related to tumor cell invasiveness) and the type of response to postoperative treatment, deserve further study with regard to these relationships, in our opinion. It is noteworthy that a combined L-EGFR and H-Beclin1 GB profile seems to identify, in our observations, a subgroup of long-surviving patients. This new disclosure might contribute to the other available data suitable for prognostic stratification of GB patients and also envisage future implications for targeted therapies.

## Figures and Tables

**Table 1:** Distribution of patient in each category, on account of clinical parameters and in each L/H-EGFR and L/H-Beclin1 protein expression group. Significant (*P* < 0.05) correlations with clinical parameters are also given for each EGFR and Beclin1 expression group, and in L-EGFR + H-Beclin1 versus all the other expression groups in regard of both these proteins. Objective Response (OR) was considered Complete Radiological Response plus Partial Radiological Response.

	L-EGFR *N*° pts	H-EGFR *N*° pts	*P*-value
KPS			
100–80	43	48	
<70	6	20	0.03
NPS			
class 1	13	7	
class 2	20	21	
class 3	10	24	
class 4	6	16	0.002
Syncronous Multifocality			
Yes	3	16	
No	46	52	0.01
Radiological Response			
Complete Response	10	9	
Partial Response (OR)	17 (27)	9 (18)	
Stable Disease	10	15	0.013
Progressive Disease	12	35	

	H-Beclin1 *N*° pts	L-Beclin1 *N*° pts	*P*-value

KPS			
100–80	51	40	
<70	7	19	0.009
Radiological Response			
Complete Response	14	5	
Partial sdResponse (OR)	19 (33)	7 (12)	
Stable Disease	14	11	0.009
Progressive Disease	11	36	

	L-EGFR + H-Beclin1	All the other GBs	*P*-value

KPS			
100–80	24	67	
<70	0	26	0.03
NPS			
class 1	9	11	
class 2	9	32	
class 3	5	29	
class 4	1	21	0.002
Syncronous Multifocality			
Yes	0	19	
No	24	74	0.002
Radiological Response			
Complete Response	13	6	
Partial Response (OR)	8 (21)	18 (24)	
Stable Disease	3	22	
Progressive Disease	0	47	0.013

**Table 2:** Clinical (Age = age at diagnosis, NPS = Neurological Performance Status, KPS = Karnofsky Performance Status), treatment (GTR = Macroscopic Gross Total Resection, B/STR = Biopsy or Sub-Total Tumor Resection, Dose RT = total dose for radiotherapy treatment, Sequential CHT = TMZ, sequentially administered after the RT-TMZ concomitant course), and biological (EGFR expression and Beclin-1 expression) prognostic factors (Kaplan-Meier method, Survival Analysis).

	*n*° pts	Progression Disease Free Survival	*P*-value	Overall Survival	*P*-value
		Median (months)		Median (months)	
Age					
>50	102	7	0.035	11	0.04
<50	15	17		18	
NPS					
class 1	20	10		15	
class 2	41	4	0.0001	18	0.0001
class 3	34	3		11	
class 4	22	1		5	
KPS					
100–80	91	9	0.001	15	0.0001
<70	26	2		5	
Extent of Surgery					
GTR	23	17	0.003	30	0.001
B/STR	94	5		10	
Dose RT					
<54 Gy	36	3	0.04	5	0.033
54–60 Gy	57	7		18	
>60 Gy	24	10		16	
Sequential CHT					
yes	61	9	0.0001	15	0.0001
no	56	1		5	
Synchronous Multifocality					
yes	19	4	0.002	7	0.001
no	98	10		15	
MGMT status					
Methylated	38	20	0.002	22	0.003
Unmethylated	45	4		5	
EGFR expression					
Low	49	14	0.002	18	0.004
High	68	5		9	
Beclin-1 expression					
High	58	12	0.001	15	0.001
Low	59	4		5	
EGFR and Beclin1 co-expression					
** L-EGFR** ** H-Beclin1**	24	22	0.001	30	0.001
Others	93	8		11	
